# Learning curves in orthopedic and trauma surgery: a systematic review

**DOI:** 10.1530/EOR-2025-0033

**Published:** 2026-09-01

**Authors:** Pierre-Alban Bouché, Charles Falkenrodt, Rémy S Nizard, Flore Devriese, Matthieu Resche-Rigon, Jules Descamps

**Affiliations:** ^1^ Université de Paris Cité, Paris, France; ^2^ Service de Chirurgie Orthopédique et Traumatologique, Hôpital Lariboisière, APHP, Paris, France; ^3^ Service de Chirurgie Orthopédique et Traumatologique, Hopitaux Universitaire de Genève, Genève, Suisse; ^4^ Service de Biostatistique et Information Médicale, Hôpital Saint-Louis, APHP, Paris, France

**Keywords:** orthopaedic, trauma, education, learning curve, models

## Abstract

**Background:**

- The process of teaching, learning and evaluating technical skills represents a challenge for surgeons. In the past, companionship has allowed the transmission of knowledge, but without evaluating its performance. Techniques in development were performed most often with limited or without any control. However, recently, a need for a change in that domain has emerged. In order to answer this important question, the number of studies interested in learning curves in medicine, particularly in orthopedic and trauma surgery, is increasing.

**Questions/purposes:**

- What are the different methods applied in order to model the learning processes for orthopedic or trauma surgical procedures?

- Does a test exist to compare the learning curves between them?

**Methods:**

- A systematic literature search of MEDLINE, CENTRAL and Embase was carried out on October 01, 2024. From each article, data regarding the design of the study, the specialty, the primary objective, the primary endpoint, the series sizes, the methods applied to model the learning curves, the use of graphs and the use of tests were retrieved.

**Results:**

- In total, 216 studies were found relevant. Most trials were single center, retrospective and publicly funded. We found seven different methods applied to evaluate learning curves: CUSUM (cumulative summation) methods, LC-CUSUM (learning curve cumulative summation), comparisons of experienced vs beginner participants, comparisons of first procedures vs others, general linear regression models, development of a score to rate the difficulty of the procedure and survival models. We did not find any application of a method that directly compares two learning curves.

**Conclusions:**

- In the orthopedic and trauma literature, seven different methods are used to model and evaluate learning curves. There is not enough evidence to conclude which approach is most appropriate to describe learning curves in orthopedics. However, the LC-CUSUM and regression models appear to be the most robust approaches. No method was found in order to compare the learning curves between them.

**Level of evidence:**

Level II, systematic review.

## Introduction

Acquisition of technical skills represents a challenge for both the teacher and the learner. The learning curves represent the acquisition of a task over time. They were developed and largely used during the Second World War for the production of weapons (1). They were then disseminated in various fields, notably in the industry and later in health (2, 3). One of the main advantages of learning curves is the association between numerical and graphical representations of the learning process. Thus, a direct application would be to compare them with each other in order to evaluate the potential of learning different techniques. Medicine became interested, especially in the 1980s, in learning curves in order to validate new techniques to be acquired by students and assess the time taken to acquire them (2, 4). Several approaches to describe and model them have been developed in clinical epidemiology (5). One of the biggest difficulties in learning curves is to model skill acquisition, which varies considerably across domains. In surgery, learning curves have been increasingly used as an objective tool to assess technical skill acquisition, both for trainees and for the introduction of new procedures (6). Before granting autonomy to a junior surgeon, an objective measure of skill acquisition is highly desirable, as the traditional apprenticeship model, largely based on subjective assessment, has well-recognized limitations.

More recently, the development of minimally invasive techniques and the introduction of new technologies, notably robotics, have further increased the use of learning curves as an evaluation criterion (6).

We focused on learning curves for orthopedic and trauma surgical procedures to determine whether a specific approach provides a better framework for their evaluation (7). To this end, we performed a systematic review of the different methods used to model learning curves in this field. To our knowledge, no standardized method to compare different learning curves has been described, presumably due to the critical difficulties in modeling them and in developing appropriate comparison tests (5). The second aim of this study was therefore to identify available methods to compare learning curves with one another (8).

## Search strategy and criteria

### Eligibility criteria

Studies were included if they reported on the learning curve of orthopedic and trauma surgical procedures and if the article was a clinical research study. No restriction on language or date of publication was applied.

### Information sources

For this review, studies were identified by searching in electronic databases (MEDLINE, CENTRAL and Embase). Initial search was run on October 01, 2024. Finally, reference lists of systematic reviews and meta-analyses were systematically screened for any additional references.

### Search

We used the following search terms:

((((learning curve[MeSH Terms]) OR (learning curves[MeSH Terms])) OR (learning curve[Title])) OR (learning curves[Title])) AND (((orthopedic[MeSH Terms]) OR (orthopedic procedure[MeSH Terms])) OR (traumatology[MeSH Terms])).

### Study selection

Two senior orthopedic surgeons independently screened all retrieved studies based on title and abstract, followed by full-text review whenever necessary to assess eligibility. Disagreements were resolved by a third reviewer and by contacting the corresponding author in case of persisting doubt. All eligible studies were then extracted, and further selection was performed based on the full text by the same author.

### Data collection process and items

A data extraction sheet was developed by all authors. It was pilot-tested on five studies and refined. All selected studies were then reviewed independently by one author. For the review, we collected the following data from each article: general study information (date of publication, country, authors, title and funding sources), specialty, type of intervention, design (retrospective or prospective) of the study, primary objective of the study, primary endpoint of the study, series size, method used to model the learning curves and test applied if learning curves were compared. Sub-specialties were classified into elective orthopedic procedures (arthroplasty, spine, arthroscopy, foot surgery, hip preservation surgery, pediatric surgery and tumor surgery) and traumatology, which was analyzed as a distinct category. We also collected the type of the learning curves graphically represented.

### Summary measures

Binary outcomes are reported as number and percentage. Continuous outcomes are presented with median and min and max values.

### Patient involvement

Patients were not involved in any aspect of the study design, conduct or development of the research question or outcome measures. This study is a systematic review of the existing published research; therefore, there was no active patient recruitment for data collection.

## Results

### Selection and general characteristics

**Figure 1:**
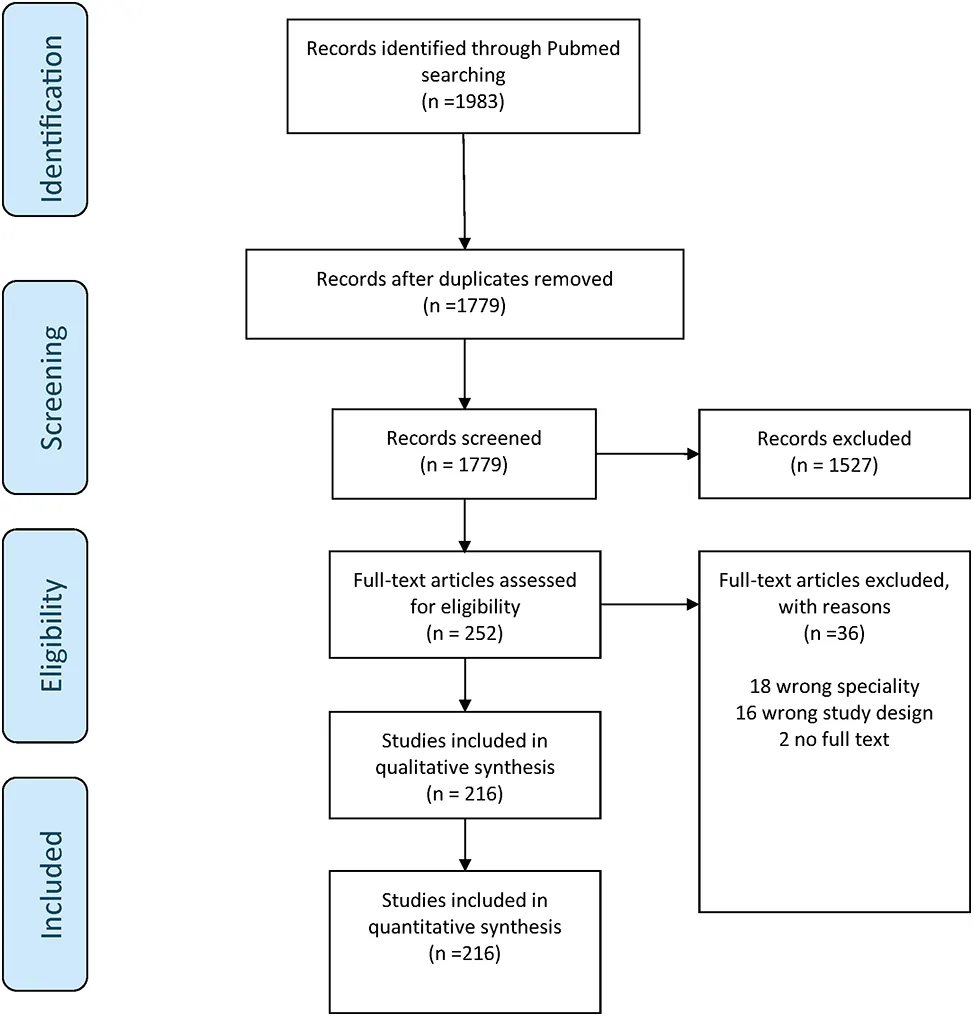
This PRISMA flow chart shows the selection process of the review of learning curves in orthopedic and trauma surgery.

Our search initially retrieved 1,983 records. Two hundred and four duplicated articles were found; thus, the titles and abstracts of 1,779 studies were reviewed. Two hundred fifty-two reports were included in the full-text review to assess eligibility. Finally, 216 studies met our inclusion criteria and were eligible for inclusion in the review (Fig. 1). Publication years ranged from 1997 to 2024, with a recent increase in the number of publications. Most trials were run in a single center and funded by public grants (respectively, 81.5 and 92.6%). Studies were performed in 34 different countries in 5 different continents. Europe and North America were the most represented continents (32.4 and 28.7%, respectively). The design was retrospective in 148 (68.5%) studies. Sub-specialties of interest were arthroplasty in 99 (45.8%) studies, spine in 51 (23.6%), arthroscopy in 31 (14.4%), foot surgery in 15 (6.9%), traumatology in 14 (6.5%), hip preservation surgery in 3 (1.4%), pediatric surgery in 2 (0.9%) and tumor in 1 (0.5%). The mean impact factor of the studies was 2.6 (1.3). The characteristics of the trials are detailed in Table 1, and the details of each trial are presented in Appendix A (see section on Supplementary materials given at the end of the article).

**Table 1:** Characteristics of the included studies. Data are presented as *n* (%) or as mean (Q1–Q3).

Variables	Values
Funding	
Private	16 (7.4%)
Public	200 (92.6%)
Continent	
Asia	60 (27.8%)
Europe	70 (32.4%)
International	7 (3.2%)
North America	62 (28.7%)
Oceania	14 (6.5%)
South America	3 (1.4%)
Number of centers	
Monocenter	176 (81.5%)
Multicenter	40 (18.5%)
Mean impact factor (*n* = 216)	2.6 (1.6–3.0)
Sub-specialties	
Arthroplasty	99 (45.8%)
Arthroscopy	31 (14.4%)
Foot	15 (6.9%)
Hip preservation	3 (1.4%)
Pediatric	2 (0.9%)
Spine	51 (23.6%)
Trauma	14 (6.5%)
Tumor	1 (0.5%)
Design	
Prospective	68 (31.5%)
Retrospective	148 (68.5%)

### Models used for learning curve

The aim of the trials was the analysis of the learning curve of a new technique in 203 (94.0%) studies and for education of surgical trainees in 13 (6.0%). The median (interquartile range) number of procedures controlled per study was 118 (Q1 = 8; Q3 = 206). The median of number of participants per study was 52 (Q1 = 8; Q3 = 22.5). We found seven different approaches to analyze learning curves in orthopedic or trauma surgical procedures. The first approach was ‘comparisons of first procedures vs others (i.e. following procedures)’.This method compared the first patients operated by a surgeon with the following ones. The threshold to define ‘first patients’ is either chosen arbitrarily or to equally separate the sample of patients or procedures. For example, out of 100 procedures, the first 50 are compared to the next 50 or 4 groups of 25 procedures are sequentially created and compared. The second approach was based on the comparison of experienced versus novice surgeons. This method is close to the ‘first vs others’ approach, but rather than taking procedures over time as the unit under control, authors compare the outcomes of procedures performed by surgeons considered experienced (defined as having completed their training and surpassed the established competency threshold) to those performed by novice surgeons or surgical trainees (defined as being in the early phase of their learning curve and having not yet reached this threshold). Two methods were based on cumulative sum methods. The CUSUM approach was developed and applied by industry since the beginning of the last century to monitor procedures and detect repeated deviations from a goal in a controlled process. From the defined goal, limits are determined for a controlled or uncontrolled process. They have been used in medicine for about thirty years to monitor stable conditions (surgical procedures, mortality in a pathology and occurrence of nosocomial infection). The LC-CUSUM approach has the same properties as the CUSUM, but here, the limit is crossed when the student has acquired the required skills, in other words when it is judged that he has entered in a controlled process. Another method was the use of the survival analysis approach. This method has been used to see the survival of implants, therefore, in studies concerning prostheses. The penultimate method was based on a regression model. Six different scenarios of learning processes were described using four models: ‘plateau’ model, ‘line–line’ model, ‘line–plateau’ model and ‘line’ model. The last method was to develop a score to rate the difficulty of the procedure.

The most frequent method used was ‘comparisons of first procedures vs others’: 75 studies (31.5%) (Table 2). A graph was associated in 148 (68.5%) studies. Most of the times, plots were in a regression curve (51 (34.5%) studies).

**Table 2:** Characteristics of methods used for learning curve comparisons. Data are presented as *n* (%) or as median (Q1–Q3).

Parameters	Values
Aim	
Education	13 (6.0%)
Technique	203 (94.0%)
No. of procedures (*n* = 215)	118 (60–206)
No. of participants (*n* = 191)	52 (−122.5)
Methods	
CUSUM	46 (21.3%)
Experienced vs beginners	14 (19.3%)
First versus others	75 (31.5%)
LC-CUSUM	5 (3.7%)
Regression	12 (31.0%)
Score	1 (0.5%)
Survival	6 (26.9%)
Graph	
No	68 (31.5%)
Yes	148 (68.5%)
Type of graph	
CUSUM	50 (23.1%)
LC-CUSUM	4 (2.7%)
Regression curve	51 (34.5%)
Scatter plot	38 (25.7%)
Survival curve	5 (3.4%)
Comparison	
No	61 (28.2%)
Yes	155 (71.8%)
Type of tests (if there is a comparison)	
Regression	29 (20.4%)
CUSUM	27 (19.0%)
LC-CUSUM	5 (3.5%)
Survival	5 (3.2%)
Standard tests	73 (51.4%)
ROC curve	3 (2.1%)

### Methods used for learning curve comparisons

A comparison between different learning curves was presented in 155 (71.8%) studies. In most of the cases (73 trials (51.4%), standard tests were used (*t*-test, chi-squared test, etc.) to compare surgical parameters, such as surgical duration or the rate of complications, between procedures of the two participants. In other cases, regression and CUSUM were used, respectively, in 29 (20.4%) studies and 27 studies (29.0%). All details are available in Table 2. We did not find any statistical method that directly compared the learning curves and allowed us to assess whether a student acquired the competence before another with a significant difference or whether one approach detected more quickly than another that a student has mastered the technique.

## Discussion

The main result of our study is that seven different approaches were identified in the literature: CUSUM (cumulative summation) methods, LC-CUSUM (learning curve cumulative summation), comparisons of experienced vs beginner participants, comparisons of first procedures vs others (i.e. following procedures), general linear regression models, development of a score for a specific procedure and survival models. This result was obtained after 216 studies were included among 1,983 retrieved relevant articles.

Learning curves in medicine allow us to observe when a procedure is acquired or when a person in training has mastered the procedure or the surgical technique (7, 9, 10). It is important to evaluate these curves in order to answer to patients and institutional demand to demonstrate that mastering of the technique was achieved to improve quality and safety of care. If there is still no duty of result, demonstration of a continuous process of evaluation, especially for new techniques or younger surgeons, appears mandatory for the future. This trend is confirmed by a PubMed extraction over the past 20 years, which demonstrates that the number of studies stating in their title or their abstract ‘learning curve’ has skyrocketed: from 155 in 1999 to 537 in 2009 and 8,164 in 2024, i.e. a 52.1-fold increase in 25 years.

To our knowledge and despite the craze for learning curves, no review has reported any information on the methods used. Thus, a review of the literature seemed interesting to more strictly evaluate the methods used to report learning processes.

We deliberately had chosen to search only for the word ‘learning curve’ in the title in electronic databases because this term is used in an abusive manner possibly to try to promote a study in our opinion. The choice to look at studies only in orthopedics was deliberately made in order to be homogeneous and to be able to have a reliable method in this field.

The first and most used method is ‘first vs others’. This method is easy to implement, reproducible and easily understood. However, it does not take into account the fact that a learning curve is not linear and can be composed of different phases. This notion is very well detailed in the article by Valsamis (5). The second most frequent method used is ‘experienced vs beginners’. This approach shares the advantages and limits of the approach mentioned above but increases uncertainty, as the operator is not his own witness. The CUSUM approach (11) is not adapted to an uncontrolled process with a high failure rate as a learning phase can be, and a modified version was developed to allow the monitoring of learning curves: LC-CUSUM (8, 12). The main advantage of LC-CUSUM is that it does not require a parametric model of the learning curve. Whatever the type of learning curve, only the passage in a controlled process is detected. Thus, it is easy to implement, adaptable to every operator and able to validate specifically this operator for a new technique. Furthermore, this technique can be performed in other specialties (13, 14) due to its flexibility and robustness (15). The main limit of LC-CUSUM is that it focuses on only one dimension and thus on only one characteristic of the learning process.

The design of studies using the survival analysis approach is developed to assess whether there is a difference in survival between implants placed early in the experiment against those implanted later. This approach is very specific to prostheses or medical devices with a life expectancy and represents a minority of studies. Some studies compared survival analysis and CUSUM methods to detect aseptic loosening of total hip arthroplasty (THA) (16, 17). CUSUM methods detected the underperformance of THA five and a half years before conventional survival analysis (17). Thus, CUSUM should be considered a very good alternative to standard statistical methods to detect deviance early.

Valsamis has developed a whole method concerning learning curves (4). Six different scenarios of learning curves were described using four models. This model is probably one of the most promising to analyze learning curves (18). Nevertheless, each regression model is specific to each participant and makes comparisons difficult. Moreover, this model could be difficult to apply. Finally, using a score to evaluate each procedure allows several parameters to be controlled. However, there is no validated score for each procedure and the assessment must be made by an independent assessor, not the surgeon.

To our knowledge, no study has compared the different approaches of modeling learning curves. The LC-CUSUM test seems to be the simplest, most flexible and robust technique compared with the CUSUM approach (19). LC-CUSUM depends only on what is considered to be an acceptable and unacceptable failure rate and is not influenced by the patient characteristics, but in some settings, what is considered acceptable could vary according to those characteristics. This technique could be applied whatever the type of acquisition of the student and the type of the interest procedure. Furthermore, it is easy to disseminate and it can be used in other specialties (13, 14). The second interest of this review was to detect whether a method was available to compare different learning curves between them. We did not find any test directly comparing learning curves or allowing us to detect whether a student acquired the competence before another with a significant difference or whether a technique had a significantly quicker (or improved) learning curve. In addition, no method exists to compare different learning curves. Different surgical techniques constantly appear and evolve, several authors will want to compare the learning curves of these new techniques against the older in order to show that they will not require a too long or chaotic learning process. Thus, it appears mandatory to develop approaches to compare these curves with a valid method.

This systematic review has several limitations that should be acknowledged. Regarding the methodological limitations of the review itself, no formal risk-of-bias assessment was performed for each included study, which restricts the appraisal of the quality and reliability of the underlying data. In addition, by focusing exclusively on orthopedic and trauma surgery for consistency, the findings may have limited generalizability to other surgical specialties.

Regarding the limitations related to the evidence derived from the included studies, the marked heterogeneity in study designs, outcome measures and definitions of the learning curve substantially complicated data synthesis and precluded any meta-analysis. This heterogeneity reflects a broader issue in the field: the absence of standardized methodology and consensual definitions, which undermines the comparability of results across studies and highlights the urgent need for clear methodological guidelines in the reporting of surgical learning curves.

## Conclusions

In the orthopedic and trauma literature, seven different methods are used to describe learning curves. There is not enough evidence to conclude which approach is most appropriate to describe learning curves in orthopedics. However, the LC-CUSUM and regression models appear to be the most robust approaches. No method was found to compare learning curves between them.

## Supplementary materials



## ICMJE Statement of Interest

The authors declare that there is no conflict of interest that could be perceived as prejudicing the impartiality of the work reported.

## Funding Statement

The researchers did not receive any funding to conduct this study.

## Data availability

Available on request.

## Ethics approval

Not applicable. This is a systematic review.
