# Supplementary material for: Learning curves in orthopedic and trauma surgery: a systematic review
Source: EFORT Open Rev. 2026 Sep 1;11(9):949–54. doi: 10.1530/EOR-2025-0033 (PMC13548107; doi:10.1530/EOR-2025-0033)
Supplement: Supplementary file 1 [file EOR-2025-0033_supplementary_materials.pdf]

Appendix A. Details of each trial:

Study	Author	Date of publication	Continent	Sub-speciality	Impact factor	Design	Number of center
Arthroscopic Latarjet procedure with double-button fixation: short-term complications and learning curve analysis	Bonnevialle	01.01.2018	Europe	Arthroscopy	2,9	Prospective	multicenter
102 lumbar pedicle subtraction osteotomies: one surgeonâ€™s learning curve	Bourghli	30.01.2018	Europe	Spine	2,6	Retrospective	monocenter
Learning curves in Motec total wrist arthroplasty: an international cohort study	Brown	08.09.2023	International	Arthroplasty	2	Retrospective	multicenter
The direct anterior minimal invasive approach in total hip replacement: a prospective departmental study on the learning curve	Brun	01.03.2018	Europe	Arthroplasty	1,3	Prospective	monocenter
The learning curve for a novel, fluoroscopyâ€based roboticâ€assisted total hip arthroplasty system	Buchan	02.04.2023	North america	Arthroplasty	2,3	Retrospective	multicenter
Defining the Learning Curve of Anterior Total Hip Arthroplasty After Fellowship-specific Training	Burnham	01.01.2022	North america	Arthroplasty		Retrospective	multicenter
Analysis of surgeon experience and impact of comorbidities on early discharge after mini-open transforaminal lumbar interbody fusion	Chan	01.07.2016	North america	Spine	0,2	Retrospective	monocenter
Learning Curve for a Dual Attending Surgeon Strategy in Posterior Spinal Fusion	Chan	01.06.2021	Asia	Spine	2,7	Retrospective	monocenter
A learning curve of total knee arthroplasty (TKA) based on surgical volume analysis	Cheng	08.06.2010	Asia	Arthroplasty	3,5	Retrospective	multicenter
Analysis of learning curve of minimally invasive total knee arthroplasty: A single surgeonâ€™s experience with 4017 cases over a 9-year period	Cheng	01.07.2019	Asia	Arthroplasty	1,9	Retrospective	monocenter
Total knee arthroplasty using patient-specific guides: Is there a learning curve?	Chinnappa	03.03.2015	Oceania	Arthroplasty	1,6	Retrospective	monocenter
Clinical Outcomes and Complications during the Learning Curve for Reverse Total Shoulder Arthroplasty: An Analysis of the First 40 Cases	Cho	01.04.2017	Asia	Arthroplasty	1,9	Retrospective	monocenter
The Total Ankle Arthroplasty Learning Curve With Third-Generation Implants: A Single Surgeonâ€™s Experience	Clement	01.08.2013	North america	Foot	1,8	Retrospective	monocenter
Navigation Reduces the Learning Curve in Resurfacing Total Hip Arthroplasty	Cobb	01.10.2007	Europe	Arthroplasty	4,4	Prospective	monocenter
Construct Validity, Assessment of the Learning Curve, and Experience of Using a Low-Cost Arthroscopic Surgical Simulator	ColaÃ§o	01.01.2016	Europe	Arthroscopy	2,6	Prospective	multicenter
Comparison of arthroscopic and open Latarjet with a learning curve analysis	Cunningham	12.12.2015	Europe	Arthroscopy	3,3	Retrospective	monocenter
Learning curve of Persona Partial Knee (PPK) arthroplasty: a clinical trial	Dâ€™Ambrosi	19.01.2024	Europe	Arthroplasty	2,2	Prospective	monocenter
Junior surgeon learning curve for performing digital planning of a first-line uncemented total hip prothesis	Cypel	17.05.2024	Europe	Arthroplasty	1,4	Prospective	monocenter
Learning curve in tissue sparing total hip replacement: comparison between different approaches	Dâ€™Arrigo	31.01.2009	Europe	Arthroplasty	1,6	Prospective	monocenter
Value of the cumulative sum test for the assessment of a learning curve: Application to the introduction of patient-specific instrumentation for total knee arthroplasty in an academic department	De Gori	16.03.2017	Europe	Arthroplasty	1,6	Prospective	monocenter

What Is the Learning Curve for the Anterior Approach for Total Hip Arthroplasty?	de Steiger	22.09.2015	Oceania	Arthroplasty	4,4	Retrospective	multicenter
Learning curve and short-term clinical outcomes of a new seven-axis robot-assisted total knee arthroplasty system: a propensity score-matched retrospective cohort study	Duan	01.06.2023	Asia	Arthroplasty	1,6	Retrospective	monocenter
Initial Learning Curve for Robot-Assisted Total Knee Arthroplasty in a Dedicated Orthopedics Center	Dragosloveanu	06.11.2023	Europe	Arthroplasty	3	Retrospective	monocenter
The Learning Curve in Hip Arthroscopy: Effect on Surgical Times in a Single-Surgeon Cohort	Dumont	01.05.2020	North america	Arthroscopy	4,4	Retrospective	monocenter
Robot-assisted knee arthroplasty: Analyzing the learning curve and initial institutional experience	Ejnisman	12.04.2024	South america	Arthroplasty	4,5	Retrospective	monocenter
Navigation improves the learning curve of transforamimal percutaneous endoscopic lumbar discectomy	Fan	03.09.2016	Asia	Spine	2	Retrospective	monocenter
What Is the Expected Learning Curve in Computer-assisted Navigation for Bone Tumor Resection?	Farfalli	25.02.2016	South america	Tumor	4,4	Retrospective	monocenter
Learning curve of junior surgeons in robot-assisted pedicle screw placement: a comparative cohort study	Feng	14.11.2023	Asia	Spine	2,6	Prospective	monocenter
The Surgical Learning Curve for Cervical Disk Replacement	Federico	01.03.2024	North america	Spine	1,6	Retrospective	monocenter
Describing the spine surgery learning curve during the first two years of independent practice	Ferguson	27.10.2021	North america	Spine	1,4	Retrospective	monocenter
Surgical skills: Can learning curves be computed from recordings of surgical activities?	Forestier	03.03.2018	Europe	Spine	2,3	Retrospective	monocenter
Learning curve of thoracic pedicle screw placement using the free-hand technique in scoliosis: how many screws needed for an apprentice?	Chen	12.11.2012	Asia	Spine	2,6	Retrospective	monocenter
Does Structured Postgraduate Training Affect the Learning Curve in Direct Anterior Total Hip Arthroplasty? A Single Surgeon's First 200 Cases	Garbarino	12.01.2021	North america	Arthroplasty	1,5	Prospective	monocenter
The arthroscopic Latarjet procedure with a posterior guided system and sutureâ€button fixation enables more precise bone block positioning in the axial plane versus anterior screw fixation	Gaujac	11.01.2024	Europe	Arthroscopy	3,3	Retrospective	monocenter
Soft tissue balancing in total knee arthroplasty using sensor-guided assessment: is there a learning curve?	Gharaibeh	20.01.2018	Oceania	Arthroplasty	1,5	Prospective	monocenter
Evaluation of the learning curve and complications in unilateral biportal endoscopic transforaminal lumbar interbody fusion: cumulative sum analysis and risk-adjusted cumulative sum analysis	Guo	01.01.2024	Asia	Spine	2,8	Retrospective	monocenter
Hip-fracture osteosynthesis training: exploring learning curves and setting proficiency standards	Gustafsson	01.07.2019	Europe	Trauma	2,5	Prospective	multicenter
Hip-fracture osteosynthesis training: exploring learning curves and setting proficiency standards	Gustafsson	01.07.2019	Europe	Trauma	2,5	Prospective	multicenter
Learning curve across 2000 thoracolumbar pedicle screw placements using O-arm navigation: technical difficulties and their solutions	Hadgaonkar	12.09.2023	Asia	Spine	2,6	Retrospective	monocenter
Arthroscopic Rotator Cuff Repair: The Learning Curve	Guttmann	01.04.2005	North america	Arthroscopy	4,4	Prospective	monocenter
Unveiling the learning curve of periacetabular osteotomy	HaertlÃ©	01.04.2024	Europe	Hip conservative	4,9	Prospective	monocenter
The surgical learning curve for percutaneous Zadek osteotomy for treatment of insertional achilles tendinopathy	Gonzalez	26.06.2024	North america	Foot	2	Retrospective	monocenter
Learning Curve With Minimally Invasive Unicompartmental Knee Arthroplasty	Hamilton	01.08.2010	North america	Arthroplasty	3,4	Retrospective	monocenter

The learning curve for the direct anterior approach for total hip arthroplasty: a single surgeonâ€™s first 500 cases	Hartford	17.02.2017	North america	Arthroplasty	1,3	Retrospective	monocenter
Residentsâ€™ Learning Curve of Total Knee Arthroplasty Based on Radiological Outcome Parameters: A Retrospective Comparative Study	Hoerlesberger	24.07.2020	Europe	Arthroplasty	3,4	Retrospective	monocenter
Tunnel positioning in anterior cruciate ligament reconstruction: how long is the learning curve?	Hohmann	19.06.2010	Oceania	Arthroscopy	3,3	Retrospective	monocenter
Retrospective clinical and Xâ€'rayâ€'based outcome analysis of a shortâ€'stem hip arthroplasty taking into account the operative learning curve over 7 years in the 3â€'year control course	Jahnke	07.07.2023	Europe	Arthroplasty	2	Retrospective	monocenter
Residentsâ€™ Learning Curve of Total Knee Arthroplasty Based on Radiological Outcome Parameters: A Retrospective Comparative Study	Hoerlesberger	01.01.2021	Europe	Arthroplasty	3,4	Retrospective	monocenter
Learning Curve and Clinical Outcomes of Ultrasonic Osteotome-based En Bloc Laminectomy for Thoracic Ossification of the Ligamentum Flavum	Hu	21.05.2023	Asia	Spine	1,8	Retrospective	monocenter
Mentoring in complex surgery: minimising the learning curve complications from peri-acetabular osteotomy	Howie	07.09.2011	Oceania	Hip conservative	2	Retrospective	monocenter
Surgeon Learning Curve for the Sinus Tarsi Approach to Intra-articular Calcaneus Fractures: Improvement With Experience	Joseph	01.12.2023	North america	Trauma	1,6	Retrospective	monocenter
What Is the Learning Curve for Robotic-assisted Pedicle Screw Placement in Spine Surgery?	Hu	19.09.2013	North america	Spine	4,4	Retrospective	monocenter
Preliminary Results and Learning Curve of the Minimally Invasive Chevron Akin Operation for Hallux Valgus	Jowett	01.06.2017	Oceania	Foot	1,3	Prospective	monocenter
Learning curves in orthopaedic surgery: a case for super-specialisation?	JAIN	01.01.2007	Europe	Pediatric	1,1	Retrospective	monocenter
Learning curve in navigated total knee replacement. A multi-centre study comparing experienced and beginner centres	Jenny	01.01.2008	Europe	Arthroplasty	1,6	Prospective	multicenter
The Surgical Learning Curve for Modified Lapidus Procedure for Hallux Valgus Deformity	Jackson	01.01.2021	North america	Foot	1,8	Retrospective	monocenter
Learning curve of robot-assisted total knee arthroplasty and its effects on implant position in Asian patients: a prospective study	Jung	12.04.2023	Asia	Arthroplasty	2,2	Prospective	monocenter
Adoption of Robotic vs Fluoroscopic Guidance in Total Hip Arthroplasty: Is Acetabular Positioning Improved in the Learning Curve?	Kamara	29.06.2016	North america	Arthroplasty	3,4	Retrospective	monocenter
Accuracy of cup orientation and learning curve of the accelerometer-based portable navigation system for total hip arthroplasty in the supine position	Kamenaga	16.04.2019	Asia	Arthroplasty	1,3	Prospective	multicenter
Learning curve for biportal endoscopic posterior cervical foraminotomy determined using the cumulative summation test	Kang	14.02.2023	Asia	Spine	2,8	Retrospective	monocenter
Hip arthroscopy learning curve: a prospective single-surgeon study	Kautzner	18.10.2017	Europe	Arthroscopy	2	Prospective	monocenter
The learning curve associated with robotic-arm assisted unicompartmental knee arthroplasty	Kayani	01.08.2018	Europe	Arthroplasty	4,9	Prospective	monocenter
Establishing the learning curve for elbow arthroscopy: surgeon and trainee perspectives on number of cases needed and optimal methods for acquiring skill	Keyt	22.04.2020	North america	Arthroscopy	2,9	Prospective	monocenter
The learning curve to ROSA: cases needed to match the surgery time between a robotic-assisted and a manual primary total knee arthroplasty	Kenanidis	27.04.2023	Europe	Arthroplasty	1,4	Retrospective	monocenter
Robotic-arm assisted total knee arthroplasty has a learning curve of seven cases for integration into the surgical workflow but no learning curve effect for accuracy of implant positioning	Kayani	17.09.2018	Europe	Arthroscopy	3,3	Prospective	monocenter

Fellowship-Trained Surgeons Experience a Learning Curve Performing Revision Total Joint Arthroplasty	Kraus	05.08.2024	North america	Arthroplasty	3,4	Retrospective	monocenter
Estimating the Learning Curve of a Novel Medical Device: Bipolar Sealer Use in Unilateral Total Knee Arthroplasties	Kuznietsova	01.03.2018	North america	Arthroplasty	4,9	Retrospective	multicenter
The Learning Curve by Operative Time for Soft Tissue Balancing in Total Knee Arthroplasty Using Electronic Sensor Technology	Lakra	01.01.2019	North america	Arthroplasty	3,4	Retrospective	monocenter
A retrospective study comparing a single surgeon's experience on manual versus robot-assisted total hip arthroplasty after the learning curve of the latter procedure â€“ A cohort study	Kong	04.04.2020	Asia	Arthroplasty	12,5	Retrospective	monocenter
Image guidance shortens the learning curve for K-wire placementâ€“ an experimental study	Kraus	14.01.2013	Europe	Trauma	2,3	Prospective	monocenter
Learning Curve and Clinical Outcomes of Minimally Invasive Transforaminal Lumbar Interbody Fusion: Our Experience in 86 Consecutive Cases	Lee	01.08.2012	Asia	Spine	2,7	Prospective	monocenter
Learning curve of basic hip arthroscopy technique: CUSUM analysis	Lee	18.10.2012	Asia	Arthroscopy	3,3	Retrospective	monocenter
Hip Arthroscopy: Analysis of a Single Surgeon's Learning Experience	Konan	01.01.2011	Europe	Arthroscopy	4,4	Prospective	monocenter
Cervical Pedicle Screw Placement Using the 'Key Slot Technique': The Feasibility and Learning Curve	Lee	01.12.2012	Asia	Spine	NA	Retrospective	monocenter
Learning Curve of Acetabular Cup Positioning in Total Hip Arthroplasty Using a Cumulative Summation Test for Learning Curve (LC-CUSUM)	Lee	01.03.2014	Asia	Arthroplasty	3,4	Retrospective	multicenter
Handheld Navigation Improves Accuracy in Direct Anterior Total Hip Replacement	Kolodychuk	15.08.2022	North america	Arthroplasty	1,5	Prospective	monocenter
Learning Curve of Internal Fixation for Nondisplaced Femoral Neck Fractures: A Cumulative Sum Analysis	Lee	01.01.2018	Asia	Trauma	1,9	Retrospective	monocenter
Assessment of the Learning Curve of Supercapsular Percutaneously Assisted Total Hip Arthroplasty in an Asian Population	Lei	07.09.2020	Asia	Arthroplasty	2,6	Retrospective	monocenter
Learning Curves in the Arthroscopic Latarjet Procedure: A Multicenter Analysis of the First 25 Cases of 5 International Surgeons	Leuzinger	01.08.2019	International	Arthroscopy	4,4	Retrospective	multicenter
The Learning Curve of Third-Generation Percutaneous Chevron and Akin Osteotomy (PECA) for Hallux Valgus	Lewis	01.01.2023	Oceania	Foot	1,3	Retrospective	monocenter
Learning Curve and Clinical Outcomes of Performing Surgery with the InterTan Intramedullary Nail in Treating Femoral Intertrochanteric Fractures	Li	19.04.2017	Asia	Trauma	2,6	Retrospective	monocenter
First 100 total hip arthroplasties performed by a young surgeon using the direct anterior approach: learning curve and complications	Li	06.10.2023	Asia	Arthroplasty	2	Retrospective	monocenter
Percutaneous endoscopic transforaminal discectomy and unilateral biportal endoscopic discectomy for lumbar disc herniation: a comparative analysis of learning curves	Li	10.05.2024	Asia	Spine	2,6	Retrospective	monocenter
Minimally invasive separation surgery for the treatment of spinal metastases with small incision and freehand pedicle screw fixation: the surgical learning curve	Lin	01.03.2022	Asia	Spine	2,2	Retrospective	monocenter
Clinical outcome and repair integrity after arthroscopic rotator cuff repair significantly improved during the surgeonâ€™s learning curve	Lim	01.10.2021	Asia	Arthroscopy	2,9	Retrospective	monocenter
The machineâ€‘vision image guided surgery system reduces fluoroscopy time, ionizing radiation and intraoperative blood loss in posterior spinal fusion for scoliosis	Lim	10.07.2023	Asia	Spine	2,6	Retrospective	monocenter
A Feasibility Study of a New Muscle Sparing â€˜â€˜C3 Dome-Hybrid Open-Door Laminoplastyâ€™â€™ A Surgical Technique, Clinical Outcome, and Learning Curve Description	Liu	01.10.2020	Asia	Spine	2,7	Retrospective	monocenter

Learning curve of tibial cortex transverse transport: a cumulative sum analysis	Liu	29.08.2023	Asia	Foot	2,8	Retrospective	monocenter
Learning curve assessment for total ankle replacement using the transfibular approach	Maccario	01.01.2021	Europe	Foot	1,9	Retrospective	multicenter
The Learning Curve Associated With Thoracoscopic Spinal Instrumentation	Lonner	01.12.2005	North america	Spine	2,7	Prospective	monocenter
Relationship between cutting errors and learning curve in computer-assisted total knee replacement	Manzotti	10.06.2009	Europe	Arthroplasty	2	Prospective	monocenter
Learning curve for active robotic total knee arthroplasty	Mahure	20.02.2021	North america	Arthroscopy	3,3	Prospective	multicenter
Surgeon Learning Curve for Pediatric Supracondylar Humerus Fractures	Liu	01.12.2011	North america	Pediatric	1,4	Retrospective	monocenter
Anterior Cervical Discectomy and Fusion: The Surgical Learning Curve	Mayo	01.10.2016	North america	Spine	2,7	Prospective	monocenter
Assessing the performance and learning curve of orthopaedic surgical trainees in primary total hip arthroplasty	McCulloch	13.07.2021	Europe	Arthroplasty	1,1	Retrospective	monocenter
The Ability of Robot-Assisted Total Knee Arthroplasty in Matching the Efficiency of Its Conventional Counterpart at an Orthopaedic Specialty Hospital	Meghpara	06.08.2022	North america	Arthroplasty	3,4	Retrospective	monocenter
Defining the Learning Curve for Hip Arthroscopy: A Threshold Analysis of the Volume-Outcomes Relationship	Mehta	01.06.2018	North america	Arthroscopy	4,2	Retrospective	multicenter
First experiences with the direct anterior approach in lateral decubitus position: learning curve and 1 year complication rate	Melman	13.02.2015	Europe	Arthroplasty	1,3	Retrospective	monocenter
Total Hip Arthroplasty Learning Curves Based on Approach for New Fellowship-trained Surgeons	Messina	01.07.2023	North america	Arthroplasty	NA	Retrospective	multicenter
Surgeon Learning Curve and Clinical Outcomes of Minimally Invasive Anterior Lumbar Interbody Fusion With Posterior Percutaneous Instrumentation	Mirza	01.12.2022	North america	Spine	NA	Retrospective	monocenter
Arthroscopic Anatomic Glenoid Reconstruction: Analysis of the Learning Curve	Moga	01.11.2018	North america	Arthroscopy	2,4	Retrospective	monocenter
Comprehensive Error Analysis for Robotic-assisted Placement of Pedicle Screws in Pediatric Spinal Deformity: The Initial Learning Curve	Morse	01.08.2021	North america	Spine	1,4	Retrospective	monocenter
Penalty for Switching Implants? Assessing the Learning Curve With a Collarless, Tapered Wedge Cementless Femoral Component	Muffly	06.03.2023	North america	Arthroplasty	1,5	Retrospective	monocenter
Minimally invasive transforaminal lumbar interbody fusion: one surgeon's learning curve	Nandyala	2014-00-00	North america	Spine	4,9	Retrospective	monocenter
Anterior minimally invasive approach for total hip replacement: five-year survivorship and learning curve	MÃ¼ller	01.03.2014	Europe	Arthroplasty	1,3	Retrospective	monocenter
Analysis of the Initial Learning Curve for Robotic-Assisted Total Knee Arthroplasty Using the ROSAÂ® Knee System	Neira	06.06.2024	Europe	Arthroplasty	3	Prospective	monocenter
The learning curve of lateral access lumbar interbody fusion in an Asian population: a prospective study	Ng	01.04.2015	Asia	Spine	2,6	Prospective	monocenter
A Surgeon That Switched to Unrestricted Kinematic Alignment with Manual Instruments Has a Short Learning Curve and Comparable Resection Accuracy and Outcomes to Those of an Experienced Surgeon	Nedopil	16.07.2022	Europe	Arthroplasty	3	Prospective	monocenter
Re-learning curve for conventional total knee arthroplasty following 30 consecutive computer-assisted total knee arthroplasties	Nicholson	01.05.2013	North america	Arthroplasty	1,5	Retrospective	monocenter
Orthostatic retractor placement reduces operating time and post-operative inflammatory response during the learning curve of anterior approach THA	Opsomer	18.10.2020	Europe	Arthroplasty	1,5	Retrospective	monocenter

The learning curve following adoption of a novel shortâ€‘stem prosthesis in total hip arthroplasty: implications on shortâ€‘term patient outcomes	Padilla	06.12.2018	North america	Arthroplasty	1,4	Retrospective	monocenter
Results of the transition from posterolateral to anterior minimally invasive approach for total hip arthroplasty	Obando	15.10.2023	South america	Arthroplasty	2,8	Retrospective	monocenter
Initial learning curve after switching to uniportal endoscopic discectomy for lumbar disc herniations	Olinger	22.02.2023	North america	Spine	2,6	Prospective	monocenter
MICA: A Learning Curve	Palmanovich	01.01.2020	Asia	Foot	1,3	Retrospective	monocenter
Learning curve of the Krackow suture technique for the repair of Achilles tendon rupture	Park	18.10.2022	Asia	Trauma	2	Retrospective	multicenter
Learning Curve for New Technology? A Nationwide Register-Based Study of 46,363 Total Knee Arthroplasties	Peltola	04.12.2013	Europe	Arthroplasty	4,4	Retrospective	multicenter
Percutaneous Transforaminal Endoscopic Discectomy Learning Curve: A CuSum Analysis	Maayan	01.11.2023	North america	Spine	2,7	Retrospective	monocenter
The Learning Curve for Adopting Hip Resurfacing Among Hip Specialists	Nunley	25.09.2009	North america	Arthroplasty	4,4	Retrospective	multicenter
A Prospective Assessment of Knee Arthroscopy Skills Between Medical Students and Residentsâ€”Simulator Exercises for Partial Meniscectomy and Analysis of Learning Curves	Putzer	01.01.2022	Europe	Arthroscopy	1,2	Prospective	monocenter
Influence of surgical experience in the learning curve of a new approach in hip replacement: anterior mini-invasive vs. standard lateral	Pogliacomi	01.05.2012	Europe	Arthroplasty	1,3	Retrospective	monocenter
Restoring hip biomechanics during the learning curve of a novice surgeon: Direct anterior approach vs posterior approach	Pujol	13.07.2021	Europe	Arthroplasty	1,5	Retrospective	monocenter
The learning curve for performing three-column osteotomies in adult spinal deformity patients: one surgeonâ€™s experience with 197 cases	Raad	10.07.2019	North america	Spine	4,9	Retrospective	monocenter
The learning curve of the direct anterior approach is 100 cases: an analysis based on 15,875 total hip arthroplasties in the Dutch Arthroplasty Register	PETERS	25.08.2022	Europe	Arthroplasty	2,5	Retrospective	multicenter
The Learning Curve Associated With Robotic-Assisted Total Hip Arthroplasty	Redmond	01.01.2015	North america	Arthroplasty	3,4	Retrospective	monocenter
The Rise of the Direct Anterior Approach: Trends, Learning Curves, and Patient Characteristics of 63,182 Primary Total Hip Arthroplasties in the Dutch Arthroplasty Register (LROI)	Rietbergen	11.01.2024	Europe	Arthroplasty	3,4	Retrospective	multicenter
Imageless Navigation in Hip Resurfacing: Avoiding Component Malposition During the Surgeon Learning Curve	Romanowski	27.11.2008	North america	Arthroplasty	4,4	Retrospective	monocenter
Spinal surgeonsâ€™ learning curve for lumbar microendoscopic discectomy: a prospective study of our first 50 and latest 10 cases	Rong	01.11.2008	Asia	Spine	7,5	Prospective	monocenter
Quantitative analysis of a spinal surgeonâ€™s learning curve for scoliosis surgery	Ryu	01.05.2016	Asia	Spine	4,9	Retrospective	monocenter
Patient-Specific Instrument Can Improve Functional and Radiographic Results during Learning Curve for Oxford Unicompartmental Knee Arthroplasty	Sanz-Ruiz	28.01.2018	Europe	Arthroplasty	1,6	Prospective	monocenter
Increased failure rates after the introduction of the TFNA proximal femoral nail for trochanteric fractures: implant related or learning curve effect?	Schmitz	11.07.2022	Europe	Trauma	2,5	Retrospective	monocenter
Displays mounted on cutting blocks reduce the learning curve in navigated total knee arthroplasty	Schnurr	09.08.2011	Europe	Arthroplasty	1,5	Prospective	monocenter
The effect of surgeonâ€™s learning curve: complications and outcome after hip arthroscopy	SchÃ¼ttler	25.05.2018	Europe	Arthroscopy	2	Retrospective	monocenter

Comparison of the Short-Term Results of the First and Last 50 Scandinavian Total Ankle Replacements: Assessment of the Learning Curve in a Consecutive Series	Schimmel	01.04.2014	Europe	Foot	2,4	Retrospective	monocenter
The "Learning Curve" of Total Hip Arthroplasty	Salai	01.01.1997	Asia	Arthroplasty	2	Retrospective	monocenter
Learning Curve of Total Hip Arthroplasty in Direct Anterior Approach without Requiring Corrective Osteotomy for Hip Dysplasia	Shen	01.05.2022	Asia	Arthroplasty	1,8	Retrospective	monocenter
Incidence of Complications During the Surgeon Learning Curve Period for Primary Total Ankle Replacement: A Systematic Review	Simonson	31.08.2015	North america	Foot	4,5	Retrospective	multicenter
Does Computer-Assisted Surgery Improve Accuracy and Decrease the Learning Curve in Hip Resurfacing? A Radiographic Analysis	Seyler	01.01.2008	North america	Arthroplasty	4,4	Prospective	monocenter
Computer navigated total knee arthroplasty: The learning curve	Smith	26.05.2010	Europe	Arthroplasty	1,5	Prospective	monocenter
Surgeon Experience Influences Robotics Learning Curve for Minimally Invasive Lumbar Fusion: A Cumulative Sum Analysis	Shahi	01.11.2023	North america	Spine	2,7	Retrospective	monocenter
Surgeon Experience Influences Robotics Learning Curve for Minimally Invasive Lumbar Fusion	Shahi	01.11.2023	North america	Spine	2,7	Retrospective	monocenter
A comparative of a single novice surgeonâ€™s direct anterior approach and posterior approach learning curves in total hip arthroplasty: a retrospective cohort study	Soza	15.06.2022	Europe	Arthroplasty	1,4	Retrospective	monocenter
Imageless robotic handpiece-assisted total knee arthroplasty: a learning curve analysis of surgical time and alignment accuracy	Savov	14.07.2021	Europe	Arthroplasty	2	Retrospective	monocenter
Does hip preservation fellowship training flatten the learning curve associated with periacetabular osteotomy?	Smith	18.09.2020	Oceania	Hip conservative	1,4	Retrospective	monocenter
Learning curve of percutaneous endoscopic transforaminal lumbar discectomy by a single surgeon	Son	01.01.2021	Asia	Spine	1,4	Retrospective	monocenter
Learning curve for imageless robotic-assisted total knee arthroplasty in non-fellowship trained joint replacement surgeons	Stegelmann	10.10.2023	North america	Arthroplasty	1,5	Retrospective	multicenter
Learning Curve for Percutaneous Endoscopic Lumbar Diskectomy in Bi-needle Technique Using Cumulative Summation Test for Learning Curve	Sun	01.09.2019	Asia	Spine	1,9	Retrospective	monocenter
The Learning Curve Associated with Robotic Total Knee Arthroplasty	Sodhi	22.11.2018	North america	Arthroplasty	1,6	Retrospective	multicenter
Clinical and radiological outcomes of robotic-assisted unicompartmental knee arthroplasty: Early lessons from the first 100 consecutive knees in 85 patients	St Marta	01.01.2022	Oceania	Arthroplasty	1,6	Prospective	monocenter
Evaluation of the Learning Curve When Transitioning From Posterolateral to Direct Anterior Hip Arthroplasty: A Consecutive Series of 1000 Cases	Stone	08.03.2018	North america	Arthroplasty	3,4	Retrospective	monocenter
The learning curve of a novel seven-axis robot-assisted total hip arthroplasty system: a randomized controlled trial	Sun	24.04.2024	Asia	Arthroplasty	2,2	Prospective	monocenter
Learning curve comparison between switching approach and switching implant in cementless short stem total hip arthroplasty	Stadler	09.09.2024	Europe	Arthroplasty	2	Retrospective	monocenter
Fluoroscopy Learning Curve in Hip Arthroscopy: A Single Surgeonâ€™s Experience	Smith	01.10.2017	North america	Arthroscopy	4,4	Retrospective	monocenter
Anterior Cruciate Ligament Reconstruction Learning Curve Study - Comparison of the First 50 Consecutive Patients of Five Orthopaedic Surgeons During a 5-Year Follow-up	Talme	30.06.2023	Europe	Arthroscopy	1,6	Retrospective	multicenter
Experimental Guide Wire Placement for Total Shoulder Arthroplasty in Glenoid Models: Higher Precision for Patient-Specific Aiming Guides Compared to Standard Technique Without Learning Curve	Schader	27.05.2024	Europe	Arthroplasty	2,2	Prospective	multicenter
Video-Assisted Thoracoscopic Surgery in Idiopathic Scoliosis - Evaluation of the Learning Curve	Son-Hing	01.03.2007	North america	Spine	2,7	Retrospective	monocenter

Robotic-arm assisted total knee arthroplasty has a learning curve of 16 cases and increased operative time of 12 min	Tay	01.11.2022	Oceania	Arthroplasty	1,5	Prospective	multicenter
Roboticâ€'arm assisted unicompartmental knee arthroplasty system has a learning curve of 11 cases and increased operating time	Tay	04.01.2022	Oceania	Arthroscopy	3,3	Retrospective	monocenter
A novel robotic surgical assistant for total knee arthroplasty has a learning curve ranging from 6 to 14 cases and exhibits high accuracy in tibial bone cuts	Thongpulsawad	07.07.2024	Asia	Arthroplasty	2,8	Retrospective	monocenter
Precise acetabular positioning, discrepancy in leg length, and hip offset using a new seven-axis robot-assisted total hip arthroplasty system requires no learning curve: a retrospective study	Tian	24.03.2023	Asia	Arthroplasty	2,8	Retrospective	monocenter
Imageless robotic-assisted total knee arthroplasty accurately restores the radiological alignment with a short learning curve: a randomized controlled trial	Thiengwittayaporn	15.08.2021	Asia	Arthroplasty	2	Prospective	monocenter
Performance and learning curve of a surgical care practitioner in completing hip aspirations	Tingle	11.09.2016	Europe	Arthroplasty	1,1	Retrospective	multicenter
The Learning Curve of Robotic-Assisted Pedicle Screw Placements Using the Cumulative Sum Analysis: A Study of the First 50 Cases at a Single Center	Torii	28.06.2022	Asia	Spine	1,2	Retrospective	monocenter
Learning curve for robotic assisted total knee arthroplasty: our experience with imageless handâ€'held Navio system	Vaidya	22.06.2022	Asia	Arthroplasty	2,2	Retrospective	monocenter
The percutaneous learning curve of 3rd generation minimally-invasive Chevron and Akin osteotomy (MICA)	Toepfer	13.07.2022	Europe	Foot	1,9	Prospective	monocenter
Identifying the learning curve for total ankle replacement using a mobile bearing prosthesis	Usuelli	01.01.2017	Europe	Foot	1,9	Retrospective	monocenter
Analyzing Learning Curve Effects: Total Ankle Replacement Design Switch and Long-Term Survival	van Es	01.01.2024	Europe	Arthroplasty	1,3	Retrospective	monocenter
Learning curve analysis of the Collum Femoris Preserving total hip surgical technique	van Oldenrijk	11.11.2013	Europe	Arthroplasty	1,3	Prospective	multicenter
Learning Curve for Surgical Treatment of Acetabular Fractures: A Retrospective Clinical Study of a Practical and Theoretical Training Course	Tosun	02.11.2017	Asia	Trauma	2,2	Retrospective	monocenter
The initial learning curve for the ROSAÂ® Knee System can be achieved in 6-11 cases for operative time and has similar 90-day complication rates with improved implant alignment compared to manual instrumentation in total knee arthroplasty	Vanlommel	01.01.2021	Europe	Arthroplasty	2	Retrospective	multicenter
The arthroscopic Latarjet: a multisurgeon learning curve analysis	Valsamis	01.04.2020	International	Arthroscopy	2,9	Retrospective	multicenter
Quantitative and individualized assessment of the learning curve in preoperative planning of the acetabular cup size in primary total hip arthroplasty	Waldstein	11.03.2021	International	Arthroplasty	2	Retrospective	multicenter
Robotâ€'assisted total knee arthroplasty is associated with a learning curve for surgical time but not for component alignment, limb alignment and gap balancing	Vermue	03.11.2020	Europe	Arthroscopy	3,3	Retrospective	multicenter
Orthopedic residentâ€™s learning curve for arthroscopic subscapularis tendon repair: short-term clinical and radiographic outcomes	VisonÃ	29.07.2017	Europe	Arthroscopy		Prospective	monocenter
A tale of two robots: Operating times and learning curves in robot-assisted lumbar fusion	Vasan	01.01.2022	Oceania	Spine	1,9	Retrospective	monocenter
Is there a learning curve in foot and ankle surgery?	Walton	15.03.2012	Europe	Foot	1,9	Prospective	monocenter
Computer-assisted preoperative planning improves the learning curve of PFNA-II in the treatment of intertrochanteric femoral fractures	Wang	07.01.2020	Asia	Trauma	2,2	Retrospective	monocenter
Early Practice All-Cause Complications for Fellowship-Trained Anterior Hip Surgeons Are Not Increased When Compared to â€œGold Standardâ€� Experienced Posterior	Wilson	12.05.2023	North america	Arthroplasty	3,4	Retrospective	monocenter

Approach Surgeons							
An evaluation of the learning curve for a complex surgical technique: the full endoscopic interlaminar approach for lumbar disc herniations	Wang	01.01.2011	International	Spine	4,9	Prospective	monocenter
Learning curves of percutaneous endoscopic lumbar discectomy in transforaminal approach at the L4/5 and L5/S1 levels: a comparative study	Wu	01.07.2016	Asia	Spine	4,7	Retrospective	monocenter
Learning curve with a new primary total knee arthroplasty implant: a multicenter perspective with more than 2000 patients	Whittaker	09.07.2018	International	Arthroplasty	1,5	Prospective	multicenter
Learning from the learning curve in total hip resurfacing: a radiographic analysis	Witjes	21.04.2009	Europe	Arthroplasty	2	Retrospective	monocenter
Unveiling the Economic Toll of Surgical Learning Curve in Elderly Hip Fractures	Yaacobi	25.07.2023	Asia	Trauma	3	Prospective	monocenter
Learning curve and initial outcomes of a novel percutaneously endoscopic-assisted total hip arthroplasty through mini bikini direct anterior approach: an observational cohort study	Wang	26.07.2024	Asia	Arthroplasty	2,8	Retrospective	monocenter
Computer navigation of the glenoid component in reverse total shoulder arthroplasty: a clinical trial to evaluate the learning curve	Wang	01.03.2020	Oceania	Arthroplasty	2,9	Prospective	monocenter
Quantification of the Learning Curve for Arthroscopic Os Trigonum Excision	Yamakado	10.11.2018	Asia	Arthroscopy	1,3	Retrospective	monocenter
Learning Curve for Minimally Invasive Scoliosis Surgery in Adolescent Idiopathic Scoliosis	Yang	01.07.2023	Asia	Spine	1,9	Retrospective	monocenter
The Learning Curve for Hip Arthroscopic Surgery: A Prospective Evaluation With 2-Year Outcomes in Patients With Femoroacetabular Impingement	You	01.10.2020	North america	Arthroscopy	2,4	Prospective	monocenter
Orthopaedic trauma surgeons and direct anterior total hip arthroplasty: evaluation of learning curve at a level I academic institution	York	03.03.2017	North america	Trauma	1,4	Retrospective	monocenter
Learning Curve for Minimally Invasive Scoliosis Surgery in Adolescent Idiopathic Scoliosis	Yang	01.07.2023	Asia	Spine	1,9	Retrospective	monocenter
The learning curve associated with imageless navigation in total knee arthroplasty	Zabat	28.02.2022	North america	Arthroplasty	2	Retrospective	monocenter
The learning curve for minimally invasive Oxford phase 3 unicompartmental knee arthroplasty: cumulative summation test for learning curve (LC-CUSUM)	Zhang	06.09.2014	Asia	Arthroplasty	2,8	Prospective	monocenter
The learning curve for minimally invasive Achilles repair using the 'lumbar puncture needle and oval forceps' technique	Zhao	03.05.2024	Asia	Foot	2,2	Retrospective	monocenter
Comparative Analysis of Learning Curve, Complexity, Psychological Stress, and Work Relative Value Units for CPT 62380 Endoscopic Lumbar Spinal Decompression vs Traditional Lumbar Spine Surgeries: A Paired Rasch Survey	Lewandrowski	25.04.2024	North america	Spine	1,7	Prospective	multicenter
Determining the learning curve for percutaneous endoscopic lumbar interbody fusion for lumbar degenerative diseases	Zhao	2023-18-193	Asia	Spine	2,8	Retrospective	monocenter
Learning curve analysis of robotic-assisted total knee arthroplasty with a Chinese surgical system	Zhang	17.11.2023	Asia	Arthroplasty	2,8	Retrospective	multicenter
Preliminary study of short-term outcomes and learning curves of robotic-assisted THA: comparison between closed platform robotic system and open platform robotic system	Zhuang	05.04.2023	Asia	Arthroplasty	2,2	Retrospective	monocenter
Learning Curve for the Two-Incision Total Hip Replacement	Archibeck	01.12.2004	North america	Arthroplasty	4,4	Prospective	multicenter
Learning Curve for Short-Stem Total HIP Arthroplasty through an Anterolateral Approach	Bischofreiter	24.04.2023	Europe	Arthroplasty	2,4	Retrospective	monocenter
Learning curves in hip fracture surgery	Bjorgul	20.02.2011	North america	Trauma	2	Prospective	monocenter

Learning curve analysis of 3D-fluoroscopy image-guided pedicle screw insertions in lumbar single-level fusion procedures	Balling	07.07.2018	Europe	Spine	2	Prospective	monocenter
Hueter Anterior Approach for Hip Resurfacing: Assessment of the Learning Curve	Benoit	01.03.2009	North america	Arthroplasty	1,4	Retrospective	monocenter
Learning Curve for Open Reduction and Internal Fixation of Displaced Intra-articular Calcaneal Fracture by Extensile Lateral Approach Using the Cumulative Summation Control Chart	Ahn	04.06.2019	Asia	Foot	2,4	Retrospective	monocenter
2nd and 3rd generation full endoscopic lumbar spine surgery: clinical safety and learning curve	Balain	17.04.2023	Europe	Spine	2,6	Prospective	monocenter
Comparative learning curves for early complications in anatomical and reverse shoulder arthroplasty	Beazley	18.02.2018	Europe	Arthroplasty	1,1	Retrospective	monocenter
Minimally invasive lumbar decompressionâ€”the surgical learning curve	Ahn	01.01.2016	North america	Spine	4,9	Retrospective	monocenter
The Learning Curve of Suprapatellar Nailing: Adoption Over Time Can Decrease Operative Time and Radiation Exposure	Allen	01.07.2020	North america	Trauma	1,6	Retrospective	monocenter
Movementâ€□based cognitive training does not significantly shorten the learning curve for acquiring arthroscopic basic skills	Alt	27.03.2024	Europe	Arthroscopy	3,3	Prospective	multicenter
Surgeons' Learning Curve of Renaissance Robotic Surgical System	BÃ¤cker	11.11.2020	North america	Spine	1,7	Retrospective	monocenter
The successful implementation of the Navio robotic technology required 29 cases	Bell	19.06.2022	North america	Arthroplasty	2,2	Prospective	monocenter
Innate Arthroscopic Skills in Medical Students and Variation in Learning Curves	Alvand	05.10.2011	Europe	Arthroscopy	4,4	Prospective	monocenter
The learning curves of a validated virtual reality hip arthroscopy simulator	Bartlett	27.01.2020	International	Arthroscopy	2	Prospective	monocenter
Validating a Global Rating Scale to Monitor Individual Resident Learning Curves During Arthroscopic Knee Meniscal Repair	Alvand	01.05.2013	Europe	Arthroscopy	4,4	Prospective	monocenter
Percutaneous Endoscopic Lumbar Discectomy Assisted by O-Arm-Based Navigation Improves the Learning Curve	Ao	10.01.2019	Asia	Spine	2,6	Prospective	monocenter
Introduction of ROSA robotic-arm system for total knee arthroplasty is associated with a minimal learning curve for operative time	Bolam	01.09.2022	Oceania	Arthroplasty	2	Prospective	monocenter
Learning curves for roboticâ€‘assisted spine surgery: an analysis of the time taken for screw insertion, robot setting, registration, and fluoroscopyre	Akazawa	26.06.2023	Asia	Spine	1,4	Retrospective	monocenter
Comparison Between Resident and Attending Surgeons as Assistants on Adolescent Idiopathic Scoliosis Surgery: No Differences in Outcomes, Complications Rate, or Pedicle Screw Placement Accuracy	Barile	01.08.2024	Europe	Spine	1,6	Retrospective	monocenter
